# Model-based conservation planning of the genetic diversity of *Phellodendron amurense* Rupr due to climate change

**DOI:** 10.1002/ece3.1133

**Published:** 2014-06-14

**Authors:** Jizhong Wan, Chunjing Wang, Jinghua Yu, Siming Nie, Shijie Han, Yuangang Zu, Changmei Chen, Shusheng Yuan, Qinggui Wang

**Affiliations:** 1 State Engineering Laboratory of Bio-Resource Eco-Utilization (Heilongjiang), Northeast Forestry UniversityHaerbin, Heilongjiang, China; 2 Institute of Applied Ecology, Chinese Academy of SciencesShenyang, Liaoning, China; 3 College of Agricultural Resource and Environment, Heilongjiang UniversityHaerbin, Heilongjiang, China

**Keywords:** Climate change, conservation areas, genetic diversity, Maxent, northeast China, Zonation

## Abstract

Climate change affects both habitat suitability and the genetic diversity of wild plants. Therefore, predicting and establishing the most effective and coherent conservation areas is essential for the conservation of genetic diversity in response to climate change. This is because genetic variance is a product not only of habitat suitability in conservation areas but also of efficient protection and management. *Phellodendron amurense* Rupr. is a tree species (family Rutaceae) that is endangered due to excessive and illegal harvesting for use in Chinese medicine. Here, we test a general computational method for the prediction of priority conservation areas (PCAs) by measuring the genetic diversity of *P. amurense* across the entirety of northeast China using a single strand repeat analysis of twenty microsatellite markers. Using computational modeling, we evaluated the geographical distribution of the species, both now and in different future climate change scenarios. Different populations were analyzed according to genetic diversity, and PCAs were identified using a spatial conservation prioritization framework. These conservation areas were optimized to account for the geographical distribution of *P. amurense* both now and in the future, to effectively promote gene flow, and to have a long period of validity. In situ and ex situ conservation, strategies for vulnerable populations were proposed. Three populations with low genetic diversity are predicted to be negatively affected by climate change, making conservation of genetic diversity challenging due to decreasing habitat suitability. Habitat suitability was important for the assessment of genetic variability in existing nature reserves, which were found to be much smaller than the proposed PCAs. Finally, a simple set of conservation measures was established through modeling. This combined molecular and computational ecology approach provides a framework for planning the protection of species endangered by climate change.

## Introduction

Climate change has had a huge impact on the genetic diversity of plant species and has damaged the habitat of wild plants, which continue to decline in large numbers, challenging the survival of some endangered species (De Oliveira et al. 2012). Previous studies have indicated that genetic diversity is positively correlated with species number and with the size of the population (Reed and Frankham 2003). Genetic diversity not only contributes to maintaining a genotype adapted to climate change, thus enhancing the adaptive fitness of the species (for example, rapid recovery after a natural, extreme warming) but also prevents a reduction in genetic diversity. As the relationship between genetic diversity and population dynamics is strong (Lammi et al. 1999), it is important for ecologists to use molecular ecology when devising appropriate conservation measures. When habitat suitability and genetic variance are under serious threat, or even facing extinction, there is a particular urgency to preserve samples representing genetic diversity using ex situ conservation measures away from the species' native range (Yan et al. 2008; Cires et al. 2013).

Although a number of studies indicate that a relationship exists between genetic diversity and climate change (Jump and Penuelas 2005; Habel et al. 2011; Dubey et al. 2013), there are relatively few studies addressing how to protect genetic diversity negatively affected by climate change. When conservation issues are addressed, the feasibility and operability of the conservation plans are typically poor and often require the immediate resolution of a problem, including validating protected species, determining conservation sites, and selecting protection areas (Eken et al. 2004; Tsianou et al. 2013). Therefore, there is an urgent need for a practical solution to the conservation of species affected by changes in genetic diversity (Frankham 2010; Van Zonneveld et al. 2012).

*Phellodendron amurense* is an endangered deciduous plant used for medicinal purposes (family Rutaceae), native to China, Korea, and Japan (Azad et al. 2005), that is distributed in temperate broad-leaved mixed forests. Due to excessive and illegal harvesting for use in traditional Chinese medicine, its population in the wild has declined sharply. It is therefore under state protection (category II) in China and was added to China's Red List of Biodiversity – Higher Plant Volume (http://www.zhb.gov.cn/gkml/hbb/bgg/201309/W020130917614244055331.pdf) as a vulnerable plant. The ecological protection of *P. amurense* is therefore considered extremely urgent (Yan et al. 2008; Yu et al. 2013).

Here, we illustrate how molecular ecology and computational modeling can be used to devise short- and long-term strategies for the protection of a species in response to climate change. Planning both in situ and ex situ conservation strategies for the protection of vulnerable plant species over long time frames in response to climate change can be challenging (Frankham 2010). We evaluated the genetic diversity and habitat suitability of *P. amurense* and then devised a simple protection assessment system to determine priority conservation areas (PCAs) suitable for either in situ or ex situ conservation measures. Maxent and Zonation are two models that have been increasingly used by protected area planners and managers for species rehabilitation and habitat conservation (Leach et al. 2013). The former is used to predict the density and distribution of a species, where all pixels are regarded as a possible distribution space of maximum entropy (Phillips et al. 2006), while the latter is used to design minimum reserves for wildlife, minimizing the effective space required for conservation areas to meet protection requirements (Di Minin and Moilanen 2012). By taking this approach, we were able to develop spatial conservation plans to protect against deterioration in habitat suitability induced by climate change, with a focus on establishing conservation areas meeting the following standards: (1) the ability to adapt to current and future changes in the geographical distribution of the species (Faleiro et al. 2013); (2) efficient gene flow and species migration (Young et al. 1996); and (3) a relatively long period of conservation validity for endangered plant species not affected by climate change (Zerbe 1998).

To achieve these aims, we combined molecular labeling using microsatellite markers (simple sequence repeats [SSRs]), geographical information systems (GIS), species distribution modeling (SDM) such as Maxent, and spatial conservation planning such as Zonation to devise PCAs for *P. amurense* in response to climate change in northeast China. This comprehensive molecular and computational approach permits the establishment of rational protection zones and provides a framework for the conservation of genetic diversity in other plant species. We describe a computational framework for designing PCAs for any plant species in decline and for developing long-term management plans.

## Materials and Methods

**Figure 1:**
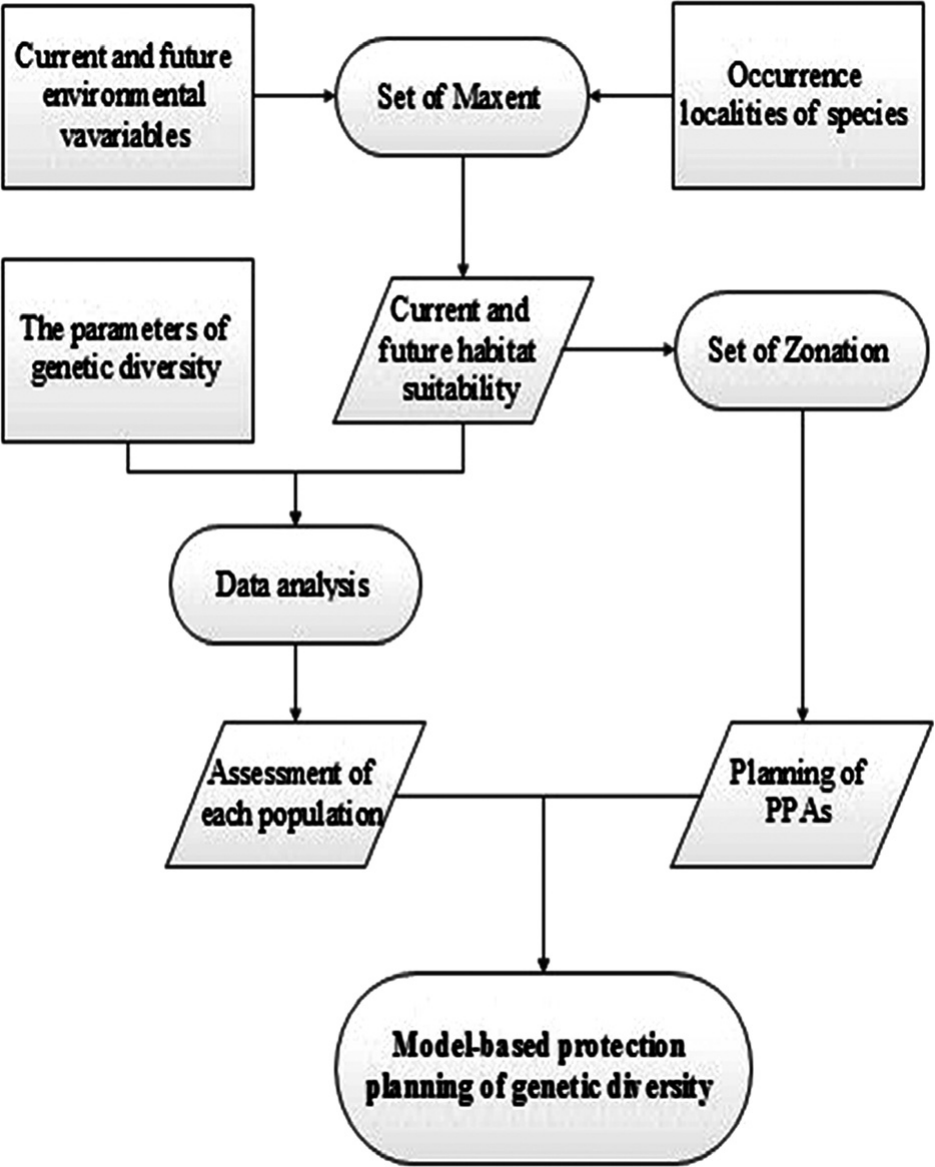
The overall scheme of the model-based conservation planning used in this study.

We devised conservation strategies to protect genetic diversity using a combination of genetic mapping and computational simulation. First, we established the associations between genetic diversity and habitat suitability to ensure that these measures provide a reasonable basis for assessing ecological processes; second, we assessed current habitat suitability and predicted future suitability under two climate change scenarios (Dubey et al. 2013); third, we used conservation planning software to plan new conservation areas resistant to the effects of climate change (Faleiro et al. 2013); finally, the operation of in situ and ex situ conservation measures were proposed for sites within the protected areas we modeled. This workflow is summarized in Figure 1 and can be applied to the assessment of any species for which genetic diversity parameters are available.

### Study area

We comprehensively surveyed the entire area of northeast China. This region includes the Heilongjiang, Jilin, and Liaoning provinces, along with parts of Inner Mongolia (Yu et al. 2011), covering an area of 1.29 × 10^6^ km^2^ (38°40′N–53°30′N, 115°05′E–135°02′E) and representing 9.8% of the total area of China (Leng et al. 2006). The geomorphological features of this area include the Changbai Mountains to the east, the Daxing'an Mountains to the north, and the Xiaoxing'an Mountains and the western mountains of Liaoning to the west. The maximum elevation of these mountain chains is below 1000 m. In the middle of these mountains lie the vast, fertile plains of northeast China, including the Sanjiang plain, the Songnen plain, and the Liaohe plain (from south to north), with a maximum elevation of 200 m. This region belongs to the temperate humid to semi-humid continental monsoon climate zone, with cold and dry winters and humid and rainy summers. Over the past 60 years, the mean annual of temperature has been 5.68°C (SD: 1.47°C). Precipitation during the summer months accounts for 50–70% of the total annual precipitation, with an annual mean of 614.9 mm (SD: 80.9 mm). This area of northeast China includes the largest area of natural forests (50.5 million ha) in the country. Forest stock in the region (3468 million m^3^) accounts for 27.8% of the national total (Fu et al. 2009).

### Data collection and utilization

Field surveys conducted over 4 years indicated that *P. amurense* was present in the forests of northeast China but that wild *P. amurense* became severely depleted over the course of the survey. The populations were very rare and mainly distributed in the eastern portion of the study area, including the Changbai Mountains, the Zhangguangcai Mountains, the Laoye Mountains, and the Xiaoxing'an Mountains.

**Figure 2:**
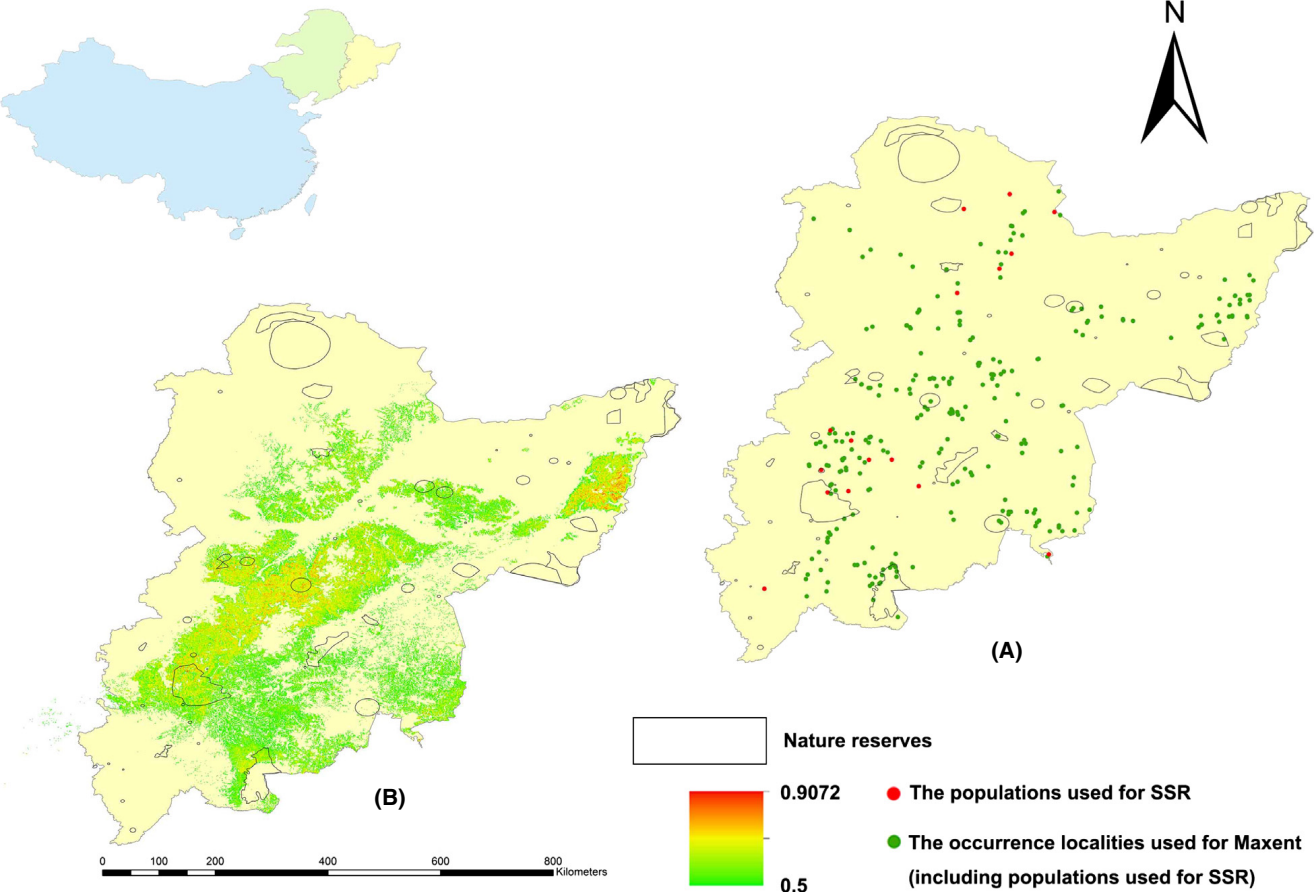
The map of (A) 315 recorded occurrence localities of *Phellodendron amurense*, and (B) the current potential geographical distribution of *P. amurense* in northeast China.

The survey period extended from 2008 to 2012. The entirety of northeast China was mapped using ArcGIS 9.2 (Esri, RedLands, CA) as the meshing tool and divided into 30 × 30 km² grids, which were surveyed systematically. Sample plots (occurrence localities) of 30 × 30 m² were selected in each study grid, and 3–8 plots were established according to the vegetation conditions of the survey area. Where possible, plots were located in the central region of the grids, and the plot's distance from the edge of the grid was never <15% of the side length of the grid. A GPS system was used to record the populations of *P. amurense* in the grid areas. In total, 315 plots were recorded as the presence points for the SDM and 1551 as the absence points for the test of model accuracy (1866 investigation plots in total). In 16 of these plots, approximately 20 samples were taken, and each sample was separated by at least 200 m, in or near the sample plots (Du et al. 2011; Li et al. 2013). A total of 279 samples were subsequently analyzed for SSR (Fig. 2 and Table S1).

The current and future data used for modeling were 0.5 arc-min (0.86 km^2^ at the equator) for the environmental layer input of the SDM. Four bioclimatic variables of 0.5-arc-min spatial resolution – mean annual temperature, mean annual precipitation, temperature seasonality, and precipitation seasonality – were downloaded from the WorldClim data base (http://www.worldclim.org) because they are considered the critical parameters for modeling the geographical distributions of plant species. The bioclimatic variables whose Pearson correlation coefficients with the other variables were between 0.8 and −0.8 were removed to eliminate the negative effect of multicollinearity on the adjustment of the SDM (Escoriza 2010; Gallagher et al. 2012). Digital elevation data (DEM; 90 m resolution) were obtained from CGIAR-CSI (http://srtm.csi.cgiar.org), and we extracted the elevation, aspect, and slope data from the DEM using ArcGIS 10.0 (Esri). Lastly, we obtained land use and land cover (LULC) data from GlobCover V2.3 (ESA Globcover Project; http://due.esrin.esa.int/globcover/; Table S2). We assumed that elevation, aspect, slope, and LULC would remain unchanged in the future.

Future bioclimatic variables were assessed using HCCPR_HADCM3 analog data (for the 2020s [2010–2039], 2050s [2040–2069], and 2080s [2070–2099]), downloaded from the International Centre for Tropical Agriculture (http://ccafs-climate.org) and used to predict how climate change would alter habitat suitability for *P. amurense* during these periods. We used the A2 and B2 emission scenarios for the future environmental layer input in Maxent. The A2 scenario represents a highly heterogeneous world with extremely unbalanced development, including self-sufficiency and local protectionism; the study region, therefore, had a large population and high growth rates. Economic development was primarily regionally orientated, with per capita economic growth and technological change being fragmented and slow. The B2 scenario presents a world committed to local solutions to economic, social, and environmental sustainability, and the global population is continuously increasing but at a rate lower than in the A2 scenario. The predicted direction of change was toward environmental conservation and the sustainable development of society. A2 differs from B2 by having larger cumulative concentrations or emissions of carbon dioxide; the result is a different pattern of climate change due to varying anthropogenic emissions of greenhouse gases and other pollutants. Therefore, A2 and B2 were used as high and low emissions scenarios, respectively (Johns et al. 2003; Jones et al. 2009; Ramirez-Villegas and Jarvis 2010). The temperature and precipitation changes in northeast China during the 21st century under the A2 and B2 scenarios are shown in Table S3 (Editorial Board of National Assessment of Climate Change 2007). The conservation map of northeast China was obtained from the World Database on Protected Areas (http://www.wdpa.org/).

### SSR analysis

In total, 279 *P. amurense* samples (5–6 leaves from each sample without plant disease or insects) from 16 populations were tested. A set of twenty simple sequence repeats (SSRs) was selected according to polymorphism (Yu et al. 2013). The total volume of each PCR reaction was 20 *μ*L, which contained 8.6 *μ*L of template DNA, 10 *μ*L of 2× PCR Mix (Boyouxinchuang Biotech, Beijing, China), 0.3 *μ*L of E00 (10 *μ*mol/L), 0.3 *μ*L of H_2_0 (10 *μ*mol/L), and 0.2 *μ*L of *Taq* polymerase. The PCR program was denaturation at 94°C for 45 sec, annealing at 50°C for 45 sec, five cycles of extension at 72°C for 45 sec, followed by a final extension step for 10 min at 72°C. The PCR products were stored at 4°C. The PCR amplifications were performed using the GeneAmp PCR 9600 System (Applied Biosystems, Foster City, CA; Yu et al. 2012).

The following genetic parameters were assessed: the average number of alleles per population (Na), the average number of effective alleles per population (Ne), the average observed heterozygosity per population (Ho), the average expected heterozygosity per population (He), the fixation index (*F*), and Shannon's index (*I*); indirect gene flow (Nm) was computed using GenAlEx (PAS) 6.5 for pairwise population differentiation (Peakall and Smouse 2006).

### Prediction and evaluation of habitat suitability

Maxent (ver.3.3.3; http://www.cs.princeton.edu/∼schapire/maxent/) was used to model the current and future potential distributions of *P. amurense* in the SDM. To do this, the function of habitat suitability with maximum entropy was estimated (Phillips and Dudík 2008; Kumar 2012), and then the geographical locations of *P. amurense* was modeled based on environmental variables (Kumar and Stohlgren 2009). Maxent chooses a probability distribution closest to a uniform distribution (i.e., the one coming from the background sample) subject to a set of constraints (averages of environmental variable values where the species is found). All pixels were regarded as the possible distribution space of maximum entropy (Slater and Michael 2012). The data of occurrence localities and randomly sampled background points are combined with climate variables to model the probability of the occurrence of the target species within each grid cell. The model used a sequential-update algorithm that iteratively picks a weight to minimize the result of regularized log loss for model adjustment and thus, to guarantee the convergence to the probability of distribution, namely, the Maxent result. Furthermore, it determines the potential areas of distribution by comparing the areas where the climate conditions of the study region are similar (Phillips and Dudík 2008).

Maxent has the following advantage: (1) it has the ability to handle low sample sizes, which drastically impact both the performance and the adjustment of SDM (Coudun and Gégout 2006; Guisan et al. 2007); (2) it is insensitive to multicollinearity (Evangelista et al. 2011), which can disturb the species-environment relationship analysis in multiple regression settings (Dormann 2007; Dormann et al. 2008); and (3) it provides the relative contribution of each variable. The advantages of Maxent enable us to accurately assess and predict habitat suitability (Adhikari et al. 2012). In this study, the occurrence probability of the potential distribution of *P. amurense* was interpreted as habitat suitability, and the distribution pixels of the wild *P. amurense* samples collected in the field surveys were treated as sample occurrence localities (Dubey et al. 2013). To visualize the Maxent results, we used the logistic output format, an estimate of probability of presence. A Maxent cell value of 1 was the highest possible habitat suitability score and that closest to 0 was the lowest.

The GPS positions (longitude and latitude) of 315 *P. amurense* plots were used as presence point inputs. We found that the set of Maxent parameters from Phillips et al. (2006) and Elith et al. (2011) are suitable for most studies, as they give rise to highly accurate SDM. Hence, we set our Maxent parameters basically consistent with those used by these scholars. Of the 315 locations, 75% were used for model training with the remaining 25% for testing. We repeated this process 10 times such as a cross-validation to maintain the observed prevalence of species. Models based on a random background across northeast China require less extrapolation. Hence, the maximum number of background points was set to 10000 according to the scope of northeast China. The convergence threshold was set to 0.0001, and auto features were used. The regularization multiplier was fixed at 1, and replicated run types were cross-validated to determine the estimates of uncertainty for the response curves, the predictions, and the area under the ROC Curve (AUC). Maximum iterations were fixed at 500, and the other values were kept at their defaults (Kumar 2012; Barrett et al. 2013). The jackknife test was used in Maxent to analyze the importance of different environmental factors (Adhikari et al. 2012). We regarded probability values (Maxent values) that were equal to or greater than the threshold value of 0.5 to indicate the presence of a species, and probability values <0.5 were considered absent (Phillips and Dudík 2008).

We used three methods to test the accuracy of the model, namely, AUC, κ, and true skill statistic (TSS; Allouche et al. 2006 for details). Receiver operating characteristic (ROC) curves were obtained and each value of the predicted results was regarded as a possible judging threshold for the calculation of its corresponding sensitivity and specificity. The ROC curve represents the relationship between sensitivity, which represents the absence of omission error (the true positive rate), with 1 – specificity, representing a commission error (the false positive rate; Phillips et al. 2006; Kumar 2012). Sensitivity was presented by the proportion of test localities where the model correctly predicts the presence of the species (1 – extrinsic omission rate). The quantity (1 – specificity) represented the proportion of all map pixels predicted to have suitable conditions for the species (Phillips et al. 2006). The precision of the model was evaluated based on the area under the ROC Curve (AUC). The greater the AUC value, the better the predictive effect of species distribution (Warren and Seifert 2011). The model was graded as follows: poor (AUC < 0.8), fair (0.8 < AUC < 0.9), good (0.9 < AUC < 0.95), or very good (0.95 < AUC < 1.0; Adhikari et al. 2012). TSS was used to forecast and compare the number of correctly classified forecasts, excluding those attributable to random guessing, to that of a hypothetical set of perfect forecasts. The κ statistic was used to measure the proportion of correctly predicted sites after accounting for the probability of chance agreement. These two measures range from −1 to +1, where +1 indicates a perfect agreement and values of ≤0 indicate a performance amounting to a random prediction, but are not affected by prevalence or size of the validation data set (Allouche et al. 2006).

### Data analysis

We extracted the current and future habitat suitability for each population and then used the current and the two future scenario values from Maxent to assess habitat suitability. The binomial fitting method was used to compare the Maxent score of each population; namely, habitat suitability with genetic parameters, such as the species number of each population, Na, Ne, *I*, Ho and He, and parameters showing high correlation coefficients with habitat suitability were extracted and turned into 3D scatter diagrams using JMP 10.0 and Origin 9.0 (Dubey et al. 2013; Vagelas et al. 2013).

### Identifying PCAs

We know that the species' genotype might change to adapt to new habitats, which would take a long time and would require the creation of a stable environment in which the species could evolve to prevent extinction. Therefore, we used the Zonation conservation planning software (http://cbig.it.helsinki.fi/software/) to develop conservation plans for maintaining genetic diversity in response to climate change. Zonation is typically used as a spatial conservation prioritization framework for large-scale conservation planning for species, but, here, we adapt the Zonation algorithm to establish protection areas for maintaining the genetic diversity of *P. amurense* across large spatial and temporal scales. The highest priorities for conservation, namely protected areas for maintaining genetic diversity, were confirmed by identifying the top-ranking cells after computation (Moilanen et al. 2012). We minimized the geographic distances between the current and future stable habitat distributions and considered the influence of the change in habitat suitability on the selection of reserves.

First, the predicted presence distribution maps of *P. amurense* in the present day and for the A2 and B2 future scenarios (in the 2020s, 2050s, and 2080s), as assessed by Maxent values ≥0.5 for each pixel (the input layers for Zonation), were used in the Zonation algorithm to simulate conservation areas in current and future climates. Our goal was to protect the entire current presence distribution of *P. amurense* from climate change. These maps of the present and the A2 and B2 scenarios (in the 2020s, 2050s, and 2080s) were regarded as the habitat suitability distributions of *P. amurense* weighted equally for the inputs of Zonation.

Second, to effectively promote gene flow, we modeled consistent and effective core areas and considered the validity of the future reserves (Faleiro et al. 2013; Li et al. 2013). Hence, we adopted the original core-area cell removal rule to minimize biological loss by only picking cells with the smallest values for the most valuable occurrences in the present and in the 2020s, 2050s, and 2080s (Leach et al. 2013). To aggregate conservation values, the distance between the current and future potential distributions was minimized based on habitat suitability. Using the original core-area cell removal rule, we set spatial priorities and computed the marginal loss of each cell, which we then used to determine if a conservation goal had been reached, that is, protecting a given proportion of the distributions of all species with a high priority ranking (Lehtomäki and Moilanen 2013). We used Moilanen et al. (2012) as the detailed sets of Zonation in our analysis.

Finally, the maps representing the distributions of habitat suitability for maintaining genetic diversity and the existing protection zones were superimposed on the zonation maps to identify and confirm the most important protection zones. In this way, we were able to evaluate the efficacy of the existing protected areas and to plan the construction of new conservation areas. We then analyzed the important existing nature reserves for maintaining genetic diversity through the computation of PCAs.

## Results

The genetic diversity of *P. amurense* was effectively evaluated. The sampled and recorded numbers and their genetic diversity parameters are shown in Table S4. Across the entire population, the sampled and recorded numbers of *P. amurense* were 5–31 and 20–75, respectively; Na values were 2.3333–5.4000; Ne was 1.7445–3.1080; Ho was 0.4775–0.7096; He was 0.3725–0.6116, and *I* values were 0.5887–1.2100. The values of *F* were −0.6289 to 0.1516, which were used to judge the degree to which the population experienced inbreeding or depression (Table S4). The Nm value was 2.089, from which we concluded that the current geographical distribution had not significantly impacted the history of gene flow among populations.

**Figure 3:**
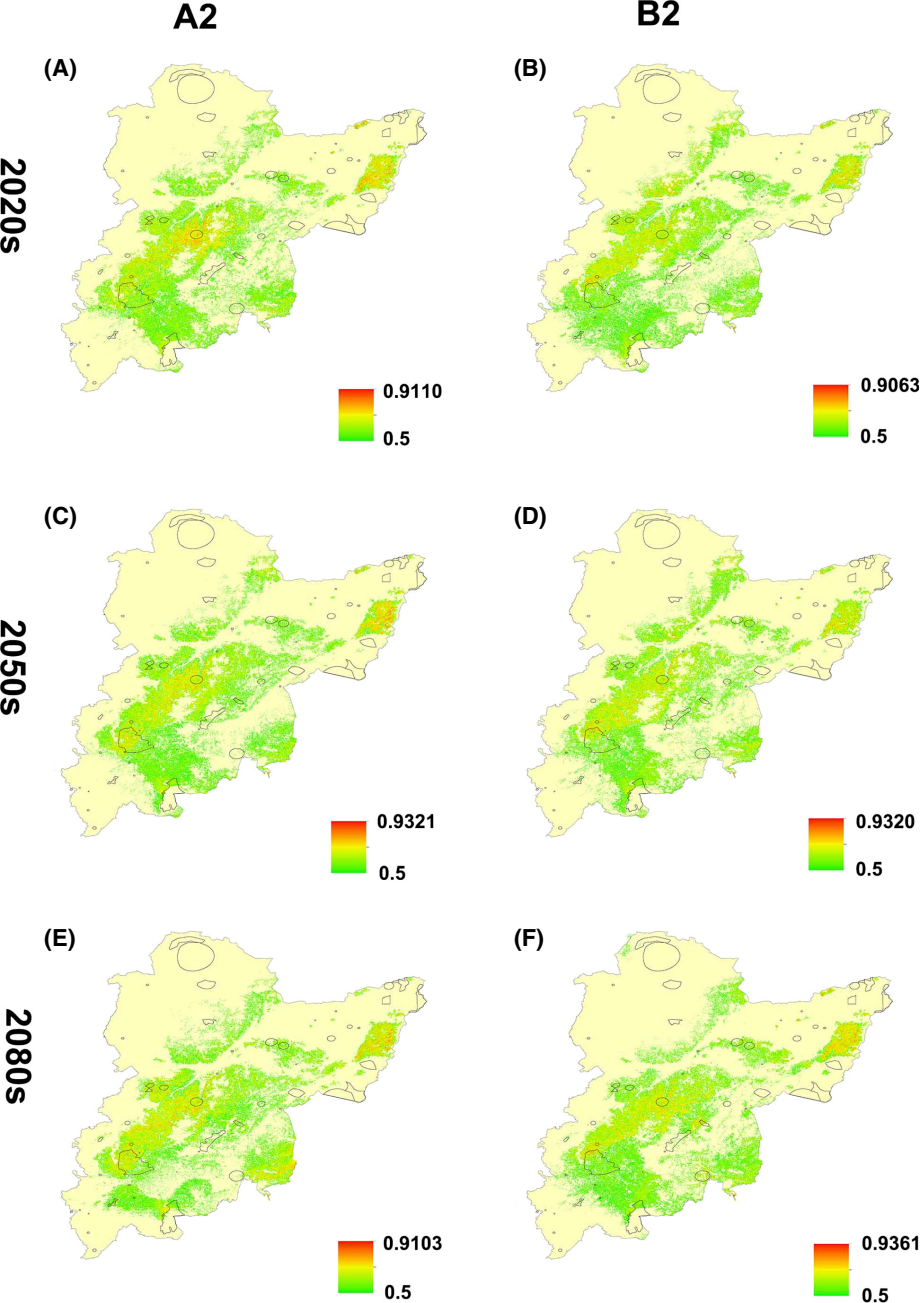
The future distribution of *Phellodendron amurense*, as predicted by modeling. This figure shows the Maxent maps using different scenarios, such as HCCPR_HADCM3 (2020s [2010–2039], 2050s [2040–2069], and 2080s [2070–2099]): A2 and B2. The color distribution from light to dark represents increasing occurrence probabilities of the species and increasing habitat suitability for *P. amurense* in the study region. (A) The habitat suitability in A2 emission scenario of 2020s; (B) B2 of 2020s; (C) A2 of 2050s; (D) B2 of 2050s; (E) A2 of 2080s; (F) B2 of 2080s.

**Figure 4:**
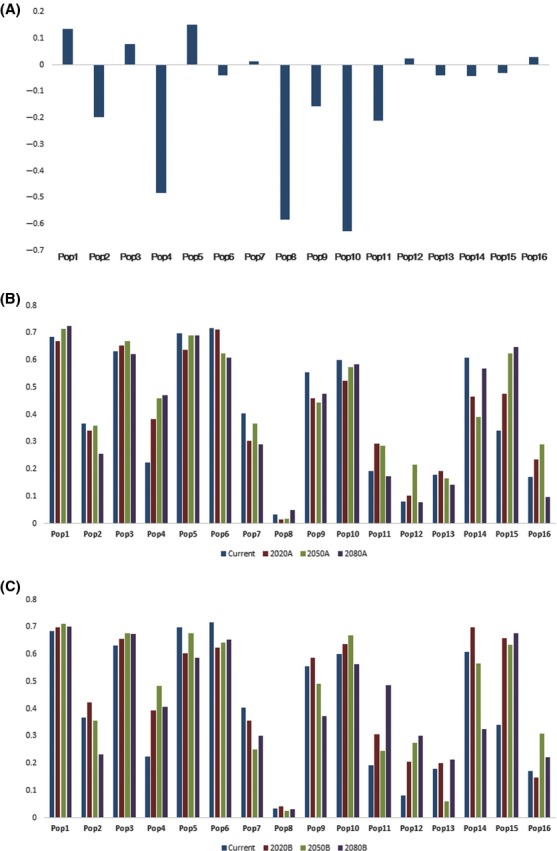
Overview of the fixation indexes of each population and the current and future habitat suitability. (A) The fixation index of each population; (B) current and future habitat suitability in A2 emission scenario; (C) B2.

Validation of the SDM showed that it demonstrated strong model performance, with AUC values >0.9 for both the training and test sets (present day: training, 0.944; test, 0.930), κ value at 0.767 and TSS value at 0.740; the variable contributions are shown in Table 1. Figures 2, 3 show the predicted habitat suitability for *P. amurense*, that is, the potential presence distributions in the present and during the 2020s, 2050s, and 2080s, with the habitat suitability for each population shown in Table S5. The areas of potential presence were mostly distributed in the eastern area of the study region, containing 73.3% of the occurrence localities, which were located in the core areas of the geographical distribution of *P. amurense* and moved slightly northward over time. We regarded the probability values (Maxent values) that were equal to or greater than the threshold value of 0.5 as indicating the presence of a species; in other words, protection is needed for populations with both low and high genetic diversity. Some populations (Pop 2, 4, 7, 8, 11, 12, 13, and 16) demonstrated continuously low habitat suitability over time (Fig. 4), which would impede the conservation of genetic diversity. These data suggest that there are serious challenges to the sustainability of *P. amurense* over the coming decades, providing the rationale for taking in situ and ex situ conservation measures.

**Table 1:** The contribution of environmental variables to Maxent

Variables	Contribution (%)
Bio12	37.6
Bio15	31.3
Bio1	1
Slope	7.5
Bio4	3
Land use and land cover (LULC)	2.5
Elevation	2.4
Aspect	0.2
Total	85.5

**Figure 5:**
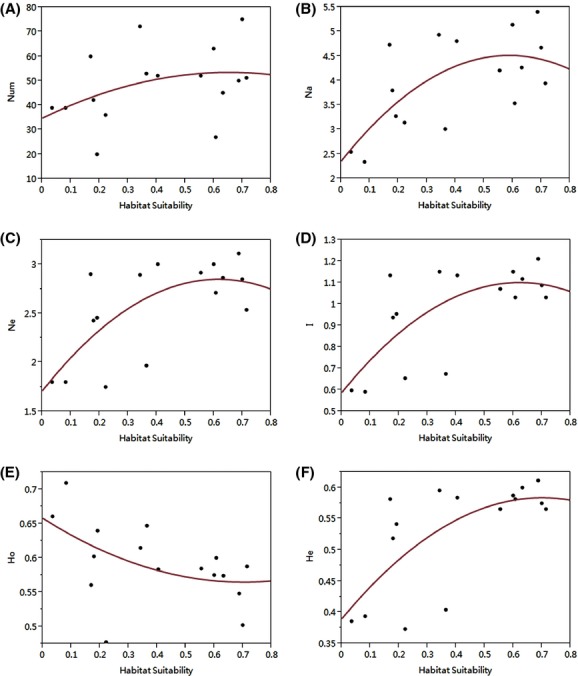
The binomial relationship between habitat suitability and genetic diversity. (A) Num, the species number of each population; (B) Na, the average number of alleles per population; (C) Ne, the effective allele number; (D) *I*, Shannon's index; (E) Ho, the average observed heterozygosity per population; (F) He, the expected heterozygosity.

**Figure 6:**
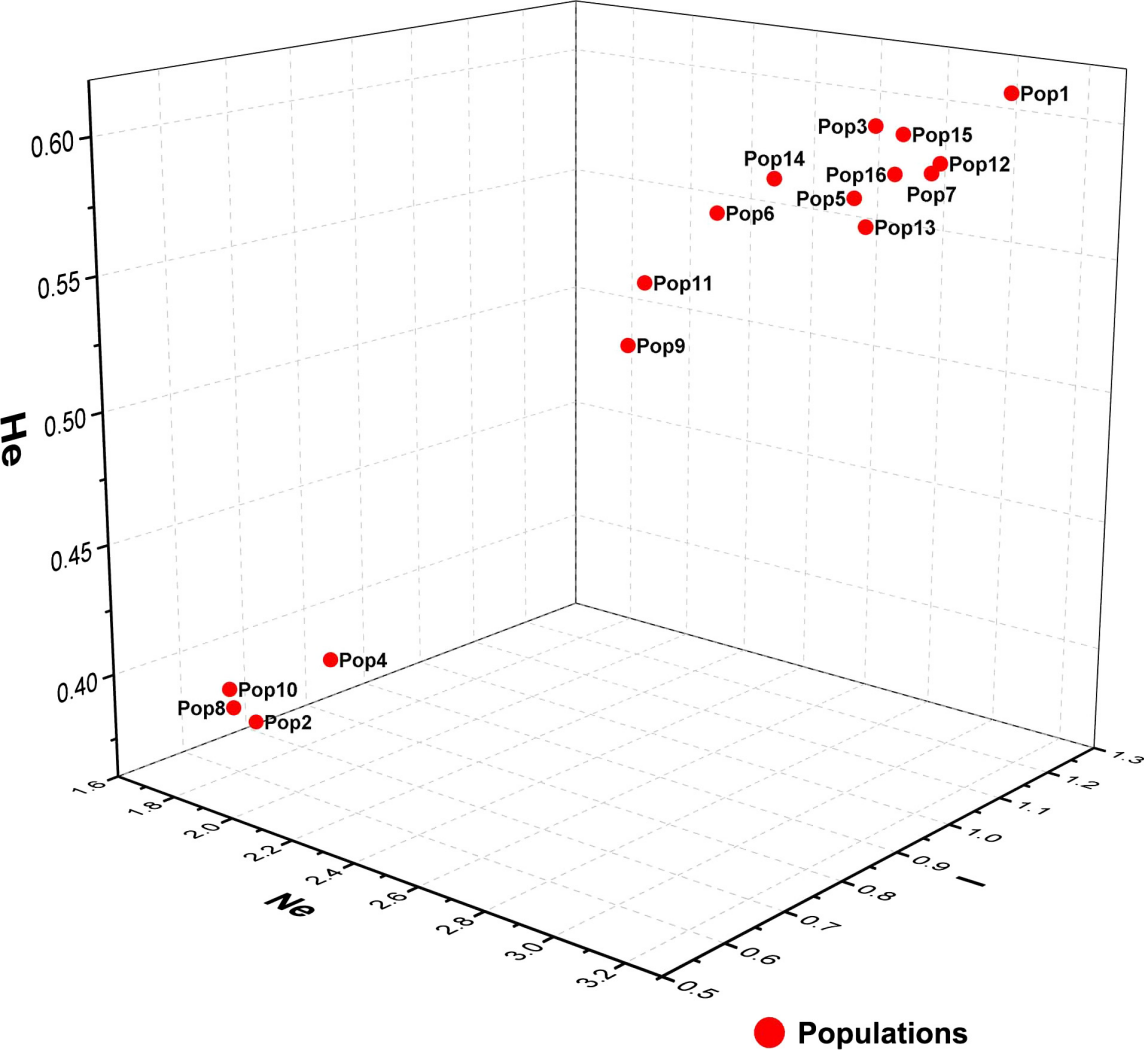
Assessment of the genetic diversity of *Phellodendron amurense* considering Ne, He, and *I*.

**Figure 7:**
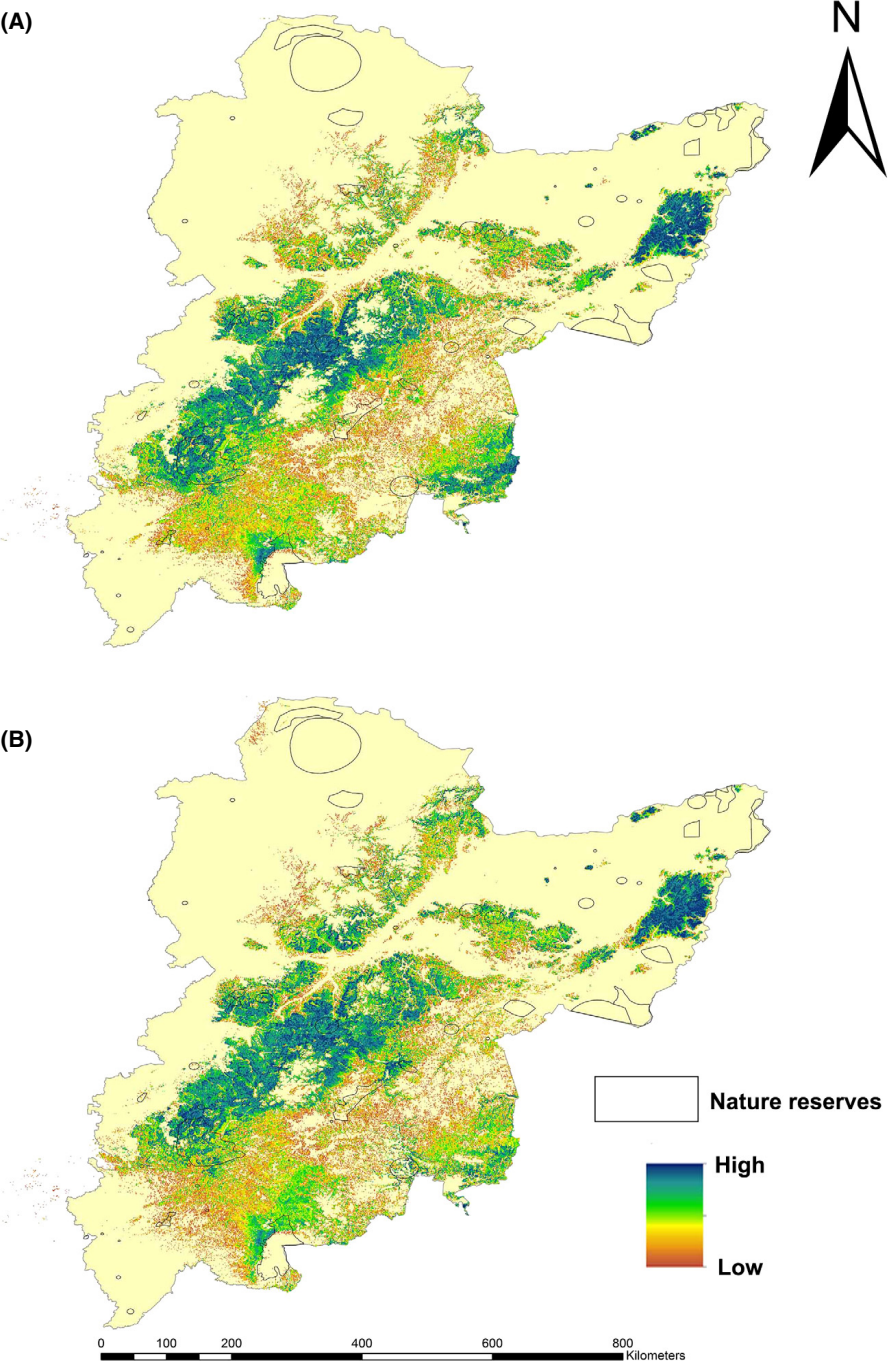
The maps of priority conservation areas for *Phellodendron amurense*. (A) and (B) were modeled using A2 and B2 emission scenarios, respectively. The color from light to dark represents increasing values of the modeled spatial conservation for the evaluation of priority conservation areas (PCAs), namely, higher priority protected levels.

Figure 5 shows there are strong relationships between He, *I*, Ne, Na, and habitat suitability. There is a positive relationship between He, *I*, Ne, Na, and habitat suitability, and, from the results of the binary fitting method, genetic diversity noticeably drops when habitat suitability falls below 0.5. Though genetic diversity will drop slightly, the overall trend is stable. The parameters such as Ne, *I*, and He that showed high correlation coefficients (*P*-values and R^2^; Table S6) with habitat suitability were extracted and turned into 3D scatter diagrams. Populations 2, 4, 8, and 10 had low genetic diversity (Fig. 6), while others had high genetic diversity. We generated two layers showing the PCAs (using Zonation software simulations) in different emissions scenarios; these layers could support the conservation of genetic diversity. The situations of PCAs are shown in Table 2. We found that the PCAs were all distributed in the eastern, northeastern, and northern areas of the study region (Fig. 7).

**Table 2:** Assessment of existing nature reserves with respect to priority conservation areas (PCAs)

Name	Type	Actual areas	Presence	PCA-A	PCA-B
Anxingshidi	Nature reserve	26.63	8.14	13.31	17.01
Huangnihe	Nature reserve	17.75	2.96	11.83	11.83
Maoershan	Nature reserve	608.69	599.82	599.82	599.82
Susu	Nature reserve	505.15	267.00	275.87	301.76
Songhuajiangsanhu	Nature reserve	4073.72	3597.41	3888.08	3767.52
Shuguang	Nature reserve	241.85	16.27	109.46	97.63
Fenghuangshan	Nature reserve	698.92	1.48	7.40	11.83
Qixinglazi	Nature reserve	409.00	320.25	360.19	372.76
Liangshui	Nature reserve	340.22	93.93	96.15	93.93
Mudanfeng	Nature reserve	224.10	16.27	164.19	180.46
Jingbohu	Nature reserve	1229.22	169.37	362.40	443.76
Songfengshan	Nature reserve	184.90	139.78	168.63	160.49
Shanhe	Nature reserve	92.45	85.05	87.27	85.79
Xidaquan	Nature reserve	8.88	0.74	0.74	0.74
Heilonggong	Nature reserve	261.08	230.02	247.03	253.68
Qingsong	Nature reserve	113.16	6.66	14.05	14.79
Jiangnanlaoyinggou	Nature reserve	22.93	17.75	22.93	22.19
Longwan	Nature reserve	193.78	4.44	153.10	88.75
Fengwugou	Nature reserve	906.75	28.84	234.45	465.21
Yanminghu	Nature reserve	10.35	2.96	2.96	2.96
Liudingshan	Nature reserve	1.48	1.48	1.48	1.48
Daomugou	Nature reserve	17.01	15.53	17.01	15.53
Xinkaihe	Nature reserve	19.23	10.35	17.01	16.27
Tiangangchaoyang	Nature reserve	56.95	54.73	55.47	56.21
Changbaishan	Nature reserve	2868.91	588.72	656.03	627.92
Total		13133.08	6279.94	7566.85	7710.33

Name represents the name of each nature reserve; Actual areas, the actual areas of the nature reserves; Presence, the area of current potential presence distributions with values of habitat suitability over 0.5; PCA-A and PCA-B represent the areas that each nature reserve can cover of the PCAs in A2 and B2 emission scenarios. Units are km^2^.

In northeast China, existing nature reserves cover considerable areas, according to the conservation gap analysis; however, the predicted areas for conservation were even larger than the existing reserve areas. Therefore, we next considered the effectiveness of the PCAs for conserving *P. amurense*, as its potential presence distributions in some counties would be expected to alter with climate change. Figure 7 shows the relationship between the existing reserves and the PCAs, from which the effectiveness of the newly established PCAs over spatial and temporal scales could be inferred. As the climate changes, some of the conservation areas would no longer be expected to protect the species as the areas of suitable habitat shift. However, some priority areas would be supported by existing reserves that would effectively protect the species. The nature reserves with the largest PCAs were Songhuajiangsanhu, Changbaishan, and Fenglin, while Nanshanchengsheshan, Laotudingzi, Dashipenggou, Daxicha, Baishilazi, and Longwan can completely protect their populations' genetic diversity, and large proportions of the potential distribution areas had high conservation contributions to the PCAs, as shown in Table 2.

## Discussion

*Phellodendron amurense* is an important tree used in Chinese medicine that is, under threat due to extensive and illegal harvesting (Yu et al. 2013). We therefore undertook 4 years of intensive and extensive field surveys to collect detailed information on the distribution and genotypic variation of *P. amurense* to devise strategies to protect the species. In doing so, we have developed a workflow using molecular ecology and computational modeling (Fig. 1) that can be used to develop conservation strategies for other endangered plant species over long time frames. The framework is illustrated using real-life genetic and field survey data for the protection of *P. amurense* over the entirety of northeast China.

Our results show that both in situ and ex situ approaches need to be considered for the effective conservation of genetic diversity in response to reduced and unstable habitat suitability (Collevatti et al. 2013). For the conservation and recovery of *P. amurense,* we suggest an increase in the number of nature reserves to protect biodiversity, in particular genetic diversity, and to establish plantations to protect its germplasm. Ex situ conservation measures, in which parts of the population are placed in a new location, could preserve the species in areas of low habitat suitability (Frankham 2010; Gong et al. 2010; Vranckx et al. 2012) and could be used to establish future environments while retaining existing genetic diversity. Although genetic diversity cannot be maintained in these areas due to the impact of climate change, it is important to allow them to shift away from their native range, that is, unsuitable habitats, to alternative, appropriate environments for culturing, in other words, ex situ conservation (Perkins et al. 2012; Cires et al. 2013; Dubey et al. 2013). In situ conservation measures, which include nature reserves and various types of scenic areas, can be used to maintain the evolution and reproductive potential of the ecological system (Jiang et al. 2013). In in situ conservation, protection in each region is increased, in this case by establishing local protected zones for *P. amurense* that conserve the natural environment. For the optimal protection of the genetic diversity of *P. amurense*, an integrated approach encompassing both ex situ and in situ conservation measures, including restoring and recovering some populations into the wild using ex situ conservation, and establishing effective evaluation systems of genetic diversity are required in China (Su et al. 2011; Cires et al. 2013). Both of these approaches have the potential to be cost saving, flexible, and to supplement existing methods of conservation. In this study, we regarded those populations with low and unstable habitat suitability as the groups most likely to require ex situ conservation methods for leaving away from native unsuitable habitats. We believe that existing in situ conservation measures would be enhanced through the adoption of the proposed ex situ conservation measures (Van der Putten et al. 2010).

### Assessment of the genetic diversity and habitat of *P. amurense*

In this study, we used three genetic parameters (Ne, He, and *I*) as assessment criteria for genetic diversity (Figs. 5, 6). Ne was used to reflect the size of the genetic variation within the population, He as a surrogate of genetic diversity within the population, and *I* as a measure of biodiversity in the ecological system (Yan et al. 2008; Van Zonneveld et al. 2012). Because one parameter alone is insufficient to fully describe genetic diversity, we used all three parameters together to identify those populations with low genetic variation that are therefore in urgent need of protection. The group with low genetic diversity includes Pop 2, 4, 8, and 10, and the other populations belong to the group with high genetic diversity.

Moreover, in this study, the value of Nm is 2.089, indicating that gene flow exists between populations (Li et al. 2013). A value of *F* close to 0 is suitable for in situ conservation because of the inbreeding between populations (an *F* value close to 1 meaning there is not one genetic exchange between populations), and values much lower than 0 are suitable for ex situ measures due to an excess of heterozygosity, which could determine the methods of protection from the perspective of gene flow (Van Zonneveld et al. 2012). Hence, we think a negative *F* value (clearly below 0) requires special attention. As shown in Figure 4A and Table S4, populations 2, 4, 8, and 10 (with clearly negative *F* values) are suitable for ex situ conservation, while populations 7, 12, and 16 (moderate *F* values) are most suited to in situ conservation.

Habitat suitability was directly associated with the occurrence probability of the species and would be expected to change with climate change (Dubey et al. 2013); Figures 2, 3 illustrate these changes, and this type of map is useful for the visualization of those sites most in need of study and protection.

Moreover, the genetic variance parameters Ne, He, and *I* have a significantly positive geospatial correlation with habitat suitability, as measured using binominal regression analysis (Fig. 5). Hence, the maintenance and conservation of habitat suitability is important for the maintenance of genetic diversity (Razgour et al. 2011). Consideration of genetic diversity alone is insufficient, however, because habitat suitability is also vital in the evaluation criteria; therefore, we need to assess the habitat suitability of species. Previous studies that have assessed the responses of species to climate change have shown that individuals move to habitats that are suitable for their maintenance (Kramer et al. 2010; Sork et al. 2010; Collevatti et al. 2011; Alsos et al. 2012; Brown and Knowles 2012). Hence, we regarded the presence of the species (i.e., Maxent values ≥0.5) as a precondition of protection of genetic diversity because the plant populations are carriers of a genotype adapted to climate change (Collevatti et al. 2011). We found that when the Maxent value was below 0.5, genetic diversity dropped precipitously (Fig. 5). We believe that when genetic diversity is too high or too low and is unstable, genetic diversity can impede population viability. As genetic diversity responds with a lag to changes in habitat suitability, an irregular trend in habitat suitability may not result in well-adapted genetic diversity. A population with a high and stable genetic diversity might be more tolerant to rapid climate change. If the change in climate is drastic, while the genotype might change until adapted to the new habitat, this would take a long time and need the creation of a stable environment in which the species can evolve to prevent extinction (in this study, we set this condition as the threshold of Maxent, that is, ≥0.5; Chevin et al. 2010; Dawson et al. 2011; Hoffmann and Sgrò 2011). Although our data showed that habitat suitability is likely to decrease, it would only require small changes to meet the requirements to protect, for example, Pop 6's genetic diversity. The habitat suitability of Pops 2, 4, 7, 8, 11, 12, 13, and 16 are still below 0.5, so ex situ conservation that we transfer these populations away from less suitable habitats to highly suitable habitats is required by default (Fig. 4). In some cases, where habitat suitability is likely to be above 0.5 in some scenarios and the population has low genetic diversity, a more adaptive approach is required. For example, for Pop 10, in situ conservation in suitable habitats could be adopted as the primary plan with ex situ conservation in unsuitable habitats as a subsidiary plan in response to environmental deterioration due to climate change. Populations with less certain future habitat suitability would be protected through in situ conservation with habitat monitoring, for instance, Pop 1, 3, 5, 6, 9, and 10. Hence, we should use ex situ conservation to transfer these populations away from less suitable habitats to highly suitable habitats. Habitat suitability needs to be considered along with different future emissions scenarios to plan in situ or ex situ conservation approaches. Clearly, populations with low genetic diversity would persistently suffer in areas of low habitat suitability (where the Maxent values are much lower than 0.5); hence, these should be subjected to in situ conservation to prevent species extinction. In the current study, we found that special attention needs to be paid to the populations with unstable habitat suitability, such as Pop 14 and 15, which need to be actively monitored by conservationists (Fig. 4; Minteer and Collins 2010; Sgro et al. 2011).

### Establishing PCAs

Although the trends in geographical species distribution are not obvious, Figure 3 indicates a clear change in the distribution of habitat suitability in a northward direction. In devising protection areas, we aimed to establish cohesive nature reserves to build breeding grounds for genetic diversity (Cadillo-Quiroz et al. 2012) and to be adaptive to shifts in potential geographical redistribution in response to climate change (Mandryk et al. 2012). We also ranked the PCAs from high to low to prioritize the protection of genetic diversity; it is more likely that *P. amurense* can colonize areas with a high rank because of their stable and high habitat suitability (Fig. 7). Nature reserves with PCAs will play a particularly important role in the prevention of the negative impacts of climate change by protecting genetic diversity and by providing stable environmental conditions to promote species evolution (genotype adapting to climate change) over long periods of time (Seaton et al. 2010). From an applied perspective, Figures 2, 3, and 7 best highlight the changing trends in the localities of habitat suitability that can be used by relevant stakeholders and decision makers to formulate policy. Genetic variance is conserved due to the positive correlation between genetic diversity and habitat suitability; therefore, those areas were regarded as the PCAs requiring in situ or ex situ conservation based on current and future presence distributions. We also applied Figure 7 for the conservation of genetic diversity in different climate change scenarios and priority protected levels. This approach provides the protection areas a period of validity of nearly 100 years, providing that the climate change scenarios are confirmed. However, it is very important to consider non-climatic factors, for example, LULC, slope, aspect, and elevation, to appropriately select the protection areas (Adhikari et al. 2012; Bertrand et al. 2012).

In this study, we also studied the relationship between our proposed protection areas and the existing conservation areas in northeast China. Although existing reserves cover a proportion of northeast China, these protected areas are far smaller than the actual and potential distribution of *P. amurense*, with the overlap being too small to be effective. Hence, reserves need to be established based on actual local situations and protection costs, which then need to be further developed into a coherent system or network (Fig. 7; Zhou and Edward Grumbine 2011). These areas would be expected to be dynamic, based on the anticipated changes in the potential distribution of the species, and some protected conservation areas would inevitably be lost over time (Essl et al. 2012), with further modifications required according to the results shown here.

With respect to the important existing nature reserves, we found those with the largest contributions to genetic diversity to be Songhuajiangsanhu, Changbaishan, and Fenglin, which could form large reserves PCA within the PCAs, while the relatively small areas of Nanshanchengsheshan, Laotudingzi, Dashipenggou, Daxicha, Baishilazi, and Longwan had complete protection of genetic diversity. Changbaishan and Fenglin are key areas for in situ and ex situ conservation and provide good experimental areas for future research (Table 2). In these reserves, the construction of botanical gardens, eco-orchards, and forest eco-stations could be promoted for in situ conservation, while enhancing breeding research, encouraging the return of wild endangered plants, evaluating growth suitability, and establishing seed banks could be used as ex situ methods (Li et al. 2002; Ren et al. 2012; Turrini and Giovannetti 2012; Braverman 2014). However, these existing areas are distant from the PCAs we identified in this study, and, although these existing nature reserves play a vital role in conservation, there is still a long way to go in the development of effective and specific conservation strategies.

Together with the fact that plant distribution is not only determined by climatic variables but also non-climatic factors such as soil and human interference (Zhang et al. 2014), these are important future research directions for planning the protection of genetic diversity.

## Conclusion

Our work addresses four main problems: (1) determining the most appropriate method (in situ or ex situ conservation) to protect target populations; (2) analyzing the nature of the relationship between genetic diversity and habitat suitability; (3) delineating protection sites and areas of in situ and ex situ conservation; and (4) incorporating the regions within existing nature reserves.

Here, a simple evaluation framework has been established for the convenient assessment of genetic diversity, based on the assessment of genetic diversity and habitat suitability, which were positively spatially correlated. This allowed us to simulate PCAs for *P. amurense,* as they were represented by Maxent values, that is, habitat suitability values. Finally, we established areas for the conservation of genetic diversity through the computation of habitat suitability and accounted for the most important ecological processes that will drive species range shifts in the future. The results of this study indicate that this simple and practical modeling method can identify PCAs, which in this case could conserve the genetic diversity of *P. amurense* with nearly 100 years of validity. For the modeling of protection areas, we considered the current and future potential geographical species distributions and changes in habitat suitability and then selected in situ or ex situ conservation approaches for populations in sites within PCAs. Ultimately, this will effectively protect genetic diversity and increase gene flow and is a methodology that can be applied to any endangered species requiring future conservation planning.

## Supporting Information

**Table S1.** Description of the sampling sites of *Phellodendron amurense* populations used for SSR over a period of 4 years.

**Table S2.** Environmental variables used.

**Table S3.** The temperature and precipitation changes in northeast China during the 21st century under the A2 and B2 scenarios.

**Table S4.** Genetic diversity statistics for *Phellodendron amurense*.

**Table S5.** The current and future habitat suitability of each population.

**Table S6.** The binomial correlation analysis between genetic parameters and habitat suitability, respectively.
