# Supplementary material for: Model-based conservation planning of the genetic diversity of Phellodendron amurense Rupr due to climate change
Source: Ecol Evol. 2014 Jun 14;4(14):2884–900. doi: 10.1002/ece3.1133 (PMC4130446; doi:10.1002/ece3.1133)
Supplement: Supplementary file 1 — Table S1. Description of the sampling sites of Phellodendron amurense populations used for SSR over a period of 4 years. [file ece30004-2884-SD1.docx]

**Table S1. Description of the sampling sites of *P. amurense* populations used for SSR over a period of four years.**

| Pop | Longitude | Latitude | Alt (m) | Locality | Region |
| --- | --- | --- | --- | --- | --- |
| Pop1 | 128.0435 | 44.1032 | 1095 | Zhangguangcailing Mountains | Huangnihe Forest Farm |
| Pop2 | 127.0621 | 44.5700 | 296.5 | Zhangguangcailing Mountains | Shuiquliu Forest Farm |
| Pop3 | 127.6792 | 44.0999 | 565.2 | Zhangguangcailing Mountains | Shangying Forest Farm |
| Pop4 | 126.9162 | 43.9387 | 357.5 | Zhangguangcailing Mountains | Xiaocheng Forest Farm |
| Pop5 | 127.0176 | 43.5786 | 502.8 | Zhangguangcailing Mountains | Kaoshan Village |
| Pop6 | 127.3509 | 43.5993 | 348.5 | Zhangguangcailing Mountains | First Deer Farm |
| Pop7 | 128.4744 | 43.6790 | 456.7 | Zhangguangcailing Mountains | Dashan Forest Farm |
| Pop8 | 127.3958 | 44.4069 | 259.9 | Zhangguangcailing Mountains | Jiudinglianhua Mountain Forest Park |
| Pop9 | 129.7633 | 47.1488 | 317 | Xiaoxinganling Mountains | Gejinhe Forest Farm |
| Pop10 | 129.9544 | 47.3893 | 236 | Xiaoxinganling Mountains | Hegang Forest Management |
| Pop11 | 129.0872 | 46.7628 | 348 | Xiaoxinganling Mountains | Qingyuan Forest Farm |
| Pop12 | 129.9271 | 48.3400 | 308 | Xiaoxinganling Mountains | Wulaga Forest Farm |
| Pop13 | 129.1943 | 48.1032 | 317 | Xiaoxinganling Mountains | Fenglin Reserve |
| Pop14 | 130.6408 | 48.0547 | 373 | Xiaoxinganling Mountains | Taipinggou Forest Farm |
| Pop15 | 130.5520 | 42.5912 | 38 | Laoyeling Mountains | Fangchuan Provincial-level Scenic Spots |
| Pop16 | 126.0089 | 42.0418 | 731 | Changbai Mountains | Zhaoyang Forest Farm |

Pop represents the codes of the identified *P. amurense* populations; Alt (m) represents the altitude of the sampling sites (The unit is m); Locality is the occurrence locality of *P. amurense* populations (most species were found in Forest Farms); Region mainly consists of mountain areas, where most of the populations appeared.
