# Supplementary material for: Model-based conservation planning of the genetic diversity of Phellodendron amurense Rupr due to climate change
Source: Ecol Evol. 2014 Jun 14;4(14):2884–900. doi: 10.1002/ece3.1133 (PMC4130446; doi:10.1002/ece3.1133)
Supplement: Supplementary file 2 — Table S2. Environmental variables used. [file ece30004-2884-SD2.docx]

**Table S2. Environmental variables used.**

| **Code** | **Environmental variables** | **Unit** |
| --- | --- | --- |
| Bio1 | Annual mean temperature | °C |
| Bio4 | Temperature seasonality | C of V |
| Bio12 | Annual precipitation | mm |
| Bio15 | Precipitation seasonality | C of V |
| LULC | Land use and land cover | 23 types |
| ELE | Elevation | m |
| SLO | Slope | ◦ |
| ASP | Aspect | ◦ |

Environmental variables were used as environmental layers for the species potential habitat distribution models by Maxent; C of V represents coefficient of variation. The range of value with 23 types of LULC is from 11 (post-flooding or irrigated croplands (or aquatic)) to 230 (No data (burnt areas, clouds and so on)) and for the detailed information, we can see GlobCover V2.3 (ESA Globcover Project; http://due.esrin.esa.int/globcover/).
