# Supplementary material for: Model-based conservation planning of the genetic diversity of Phellodendron amurense Rupr due to climate change
Source: Ecol Evol. 2014 Jun 14;4(14):2884–900. doi: 10.1002/ece3.1133 (PMC4130446; doi:10.1002/ece3.1133)
Supplement: Supplementary file 3 — Table S3. The temperature and precipitation changes in northeast China during the 21st century under the A2 and B2 scenarios. [file ece30004-2884-SD3.docx]

**Table S3**

**The temperature and precipitation changes of northeast China over the 21st century under the A2 and B2 scenarios.**

|  | Temperature (℃) | | Precipitation (%) | |
| --- | --- | --- | --- | --- |
|  | A2 | B2 | A2 | B2 |
| 2020s | 1.7 | 2.1 | 1 | 3 |
| 2050s | 3.8 | 3.4 | 5 | 8 |
| 2080s | 6.1 | 4.5 | 13 | 12 |

This table shows the added values relative to the base year 1961-1990 in the A2 and B2 emission scenarios of 2020s, 2050s and 2080s.
