# Supplementary material for: Model-based conservation planning of the genetic diversity of Phellodendron amurense Rupr due to climate change
Source: Ecol Evol. 2014 Jun 14;4(14):2884–900. doi: 10.1002/ece3.1133 (PMC4130446; doi:10.1002/ece3.1133)
Supplement: Supplementary file 4 — Table S4. Genetic diversity statistics for Phellodendron amurense. [file ece30004-2884-SD4.docx]

**Table S4. Genetic diversity statistics for *P. amurense*.**

| Pop | Num | N | Na | Ne | I | Ho | He | F |
| --- | --- | --- | --- | --- | --- | --- | --- | --- |
| Pop1 | 50 | 31 | 5.4000 | 3.1080 | 1.2101 | 0.5481 | 0.6117 | 0.1346 |
| Pop2 | 36 | 19 | 3.1333 | 1.7445 | 0.6529 | 0.4775 | 0.3725 | -0.1978 |
| Pop3 | 45 | 13 | 4.2667 | 2.8617 | 1.1178 | 0.5744 | 0.5994 | 0.0789 |
| Pop4 | 53 | 17 | 3.0000 | 1.9670 | 0.6726 | 0.6471 | 0.4037 | -0.4819 |
| Pop5 | 75 | 21 | 4.6667 | 2.8523 | 1.0866 | 0.5016 | 0.5744 | 0.1516 |
| Pop6 | 51 | 11 | 3.9333 | 2.5388 | 1.0298 | 0.5879 | 0.5653 | -0.0400 |
| Pop7 | 52 | 22 | 4.8000 | 3.0005 | 1.1336 | 0.5829 | 0.5841 | 0.0139 |
| Pop8 | 39 | 21 | 2.5333 | 1.7968 | 0.5958 | 0.6603 | 0.3856 | -0.5850 |
| Pop9 | 42 | 14 | 3.8000 | 2.4267 | 0.9378 | 0.6025 | 0.5184 | -0.1549 |
| Pop10 | 39 | 20 | 2.3333 | 1.8009 | 0.5887 | 0.7096 | 0.3936 | -0.6289 |
| Pop11 | 20 | 5 | 3.2667 | 2.4527 | 0.9518 | 0.6400 | 0.5416 | -0.2111 |
| Pop12 | 63 | 25 | 5.1333 | 2.9995 | 1.1508 | 0.5754 | 0.5868 | 0.0227 |
| Pop13 | 52 | 20 | 4.2000 | 2.9121 | 1.0700 | 0.5840 | 0.5660 | -0.0383 |
| Pop14 | 27 | 6 | 3.5333 | 2.7085 | 1.0293 | 0.6000 | 0.5815 | -0.0407 |
| Pop15 | 72 | 20 | 4.9333 | 2.8921 | 1.1509 | 0.6146 | 0.5954 | -0.0316 |
| Pop16 | 60 | 14 | 4.7333 | 2.8986 | 1.1323 | 0.5604 | 0.5818 | 0.0280 |

Pop, the codes of *P. amurense* populations; Num, the species number of each population; N, number of sampling plants; Na, the average number of alleles per population; Ne, effective allele number; I, Shannon's Information index; Ho, average observed heterozygosity per population; He, expected heterozygosity; F, the fixation index. Pop11 and Pop14 have low numbers sampled due to: (1) the characteristics of these two populations were: (a) extremely low number of individuals (b) very loose spatial pattern within each population (c) growth with a number of other species (d) serious habitat destruction so we could not collect more samples; (2) there were only a few tree of which leaves could be sampled in strict accordance with our collection method.
