# Supplementary material for: Model-based conservation planning of the genetic diversity of Phellodendron amurense Rupr due to climate change
Source: Ecol Evol. 2014 Jun 14;4(14):2884–900. doi: 10.1002/ece3.1133 (PMC4130446; doi:10.1002/ece3.1133)
Supplement: Supplementary file 5 — Table S5. The current and future habitat suitability of each population. [file ece30004-2884-SD5.docx]

**Table S5. The current and future habitat suitability of each population.**

| Pop | Current | A | | |  | | B | | | |
| --- | --- | --- | --- | --- | --- | --- | --- | --- | --- | --- |
|  |  | 2020s | 2050s | 2080s | |  | | 2020s | 2050s | 2080s |
| Pop1 | 0.6865 | 0.6691 | 0.7148 | 0.7247 | |  | | 0.6979 | 0.7118 | 0.7003 |
| Pop2 | 0.3662 | 0.3414 | 0.3580 | 0.2555 | |  | | 0.4227 | 0.3579 | 0.2324 |
| Pop3 | 0.6327 | 0.6532 | 0.6705 | 0.6217 | |  | | 0.6554 | 0.6781 | 0.6750 |
| Pop4 | 0.2233 | 0.3842 | 0.4612 | 0.4710 | |  | | 0.3926 | 0.4836 | 0.4062 |
| Pop5 | 0.6991 | 0.6370 | 0.6912 | 0.6907 | |  | | 0.6034 | 0.6763 | 0.5884 |
| Pop6 | 0.7162 | 0.7117 | 0.6248 | 0.6078 | |  | | 0.6253 | 0.6427 | 0.6525 |
| Pop7 | 0.4047 | 0.3049 | 0.3671 | 0.2910 | |  | | 0.3574 | 0.2499 | 0.3005 |
| Pop8 | 0.0339 | 0.0136 | 0.0164 | 0.0492 | |  | | 0.0414 | 0.0248 | 0.0316 |
| Pop9 | 0.5560 | 0.4609 | 0.4429 | 0.4754 | |  | | 0.5868 | 0.4930 | 0.3712 |
| Pop10 | 0.6000 | 0.5237 | 0.5736 | 0.5858 | |  | | 0.6386 | 0.6686 | 0.5640 |
| Pop11 | 0.1923 | 0.2929 | 0.2863 | 0.1741 | |  | | 0.3059 | 0.2447 | 0.4869 |
| Pop12 | 0.0816 | 0.1031 | 0.2156 | 0.0794 | |  | | 0.2045 | 0.2755 | 0.3022 |
| Pop13 | 0.1801 | 0.1920 | 0.1657 | 0.1418 | |  | | 0.2005 | 0.0606 | 0.2136 |
| Pop14 | 0.6078 | 0.4656 | 0.3898 | 0.5696 | |  | | 0.6985 | 0.5661 | 0.3238 |
| Pop15 | 0.3417 | 0.4759 | 0.6233 | 0.6490 | |  | | 0.6596 | 0.6343 | 0.6767 |
| Pop16 | 0.1709 | 0.2340 | 0.2892 | 0.0957 | |  | | 0.1466 | 0.3096 | 0.2207 |

In different emission scenarios such as HCCPR_HADCM3 (2020s (2010-2039), 2050s (2040-2069), and 2080s (2070-2099)), respectively: A2 and B2; Average represents the average values of emission scenarios such as A2 and B2, respectively.
