# Supplementary material for: Model-based conservation planning of the genetic diversity of Phellodendron amurense Rupr due to climate change
Source: Ecol Evol. 2014 Jun 14;4(14):2884–900. doi: 10.1002/ece3.1133 (PMC4130446; doi:10.1002/ece3.1133)
Supplement: Supplementary file 6 — Table S6. The binomial correlation analysis between genetic parameters and habitat suitability, respectively. [file ece30004-2884-SD6.docx]

**Table S6. The binomial correlation analysis between genetic parameters and habitat suitability, respectively.**

| Code |  | Binomial fitting method | |
| --- | --- | --- | --- |
|  |  | R^2^ | *P*-value |
| Num |  | 0.1447 | 0.362 |
| Na |  | 0.4344 | 0.0246 |
| Ne |  | 0.4927 | 0.0121 |
| I |  | 0.4919 | 0.0123 |
| Ho |  | 0.2473 | 0.1578 |
| He |  | 0.4729 | 0.0156 |

Code, the codes of genetic parameters of *P. amurense*: correlation coefficient; R²: coefficient of determination using binomial regression analysis and the binomial fitting method, respectively, *P<0.05.
